# Enhanced SIRT3 activity potentiates germinal center epigenetic modulation through α-ketoglutarate

**DOI:** 10.1016/j.jbc.2026.113552

**Published:** 2026-09-12

**Authors:** Xinyu Yang, Guoyuan Peng, Qian Hao, Kexin Liu, Zhengcao Xin, Mingming Zhang, Innocent Agida, Yong Zuo, Leng-Siew Yeap, Tianshi Wang, Jianli He, Hongsheng Tan, Jinke Cheng

**Affiliations:** 1 Shanghai Key Laboratory for Tumor Microenvironment and Inflammation, Department of Biochemistry and Molecular Cell Biology, Shanghai Jiao Tong University School of Medicine, Shanghai, China; 2 Shanghai Institute of Immunology, Shanghai Jiao Tong University School of Medicine, Shanghai, China; 3 Institute of Clinical Medicine & School of Public Health, Shanghai Jiao Tong University School of Medicine, Shanghai, China; 4 Hainan Academy of Medical Sciences, Hainan Medical University, Haikou, Hainan, China

**Keywords:** germinal center, B cells, SIRT3, glutaminolysis, α-ketoglutarate, epigenetic regulation, antibody affinity maturation

## Abstract

Germinal center (GC) B cells depend on sustained epigenetic modulation to maintain transcriptional identity and support affinity maturation; however, whether mitochondrial metabolism directly enhances this chromatin state remains unclear. Here, we show that enhanced SIRT3 activity promotes glutamine-derived α-ketoglutarate (αKG) accumulation and is associated with reduced H3K27me3 enrichment at the Bcl6 locus. Using SIRT3 K223R gain-of-function mice, metabolomic profiling, and stable isotope tracing, we show that SIRT3 activation increases glutamine-derived αKG accumulation without a uniform increase in downstream tricarboxylic acid (TCA) cycle labeling. Elevated αKG was associated with reduced H3K27me3 at tested Bcl6 regulatory regions, reinforced BCL6 and AID expression, sustained B cell proliferation, and qualitatively enhanced antibody affinity maturation. Glutamine supplementation partially phenocopied these effects, whereas inhibition of glutamine metabolism attenuated them. Importantly, sustained activation of this metabolic–epigenetic axis was associated with enhanced GC expansion accompanied by increased autoantibody levels and renal IgG deposition in a pristane-induced lupus model. Collectively, our findings indicate that enhanced SIRT3 activity potentiates a glutamine-αKG-chromatin axis that links mitochondrial glutamine metabolism to germinal center epigenetic modulation and humoral immune output.

Germinal centers generate high-affinity antibodies by coupling rapid B cell proliferation to somatic hypermutation and affinity-based selection (1, 2). This reaction depends on a specialized transcriptional state centered on BCL6, which restrains differentiation and DNA damage responses while permitting activation-induced cytidine deaminase-dependent mutation and maintenance of the germinal center program (3, 4, 5, 6, 7). Although the signaling pathways that initiate germinal center reactions are well-characterized, how metabolic programs maintain germinal center chromatin identity during repeated cycles of mutation and selection remains less clear.

Immune cell metabolism can determine cell fate by more than energy production alone. Germinal center B cells differ from extrafollicular activated B cells by showing limited glycolysis and a reliance on mitochondrial metabolism, fatty acid oxidation, and nutrient availability (8, 9, 10, 11, 12, 13). These observations establish mitochondrial metabolism as important for germinal center maintenance and affinity selection, but they do not define whether mitochondria provide instructive epigenetic signals that actively stabilize germinal center identity.

Glutamine is a versatile carbon and nitrogen source for biosynthesis, redox control and anaplerosis. Its derivative α-ketoglutarate is also an obligate cofactor for Jumonji-domain histone demethylases and TET family DNA demethylases, linking carbon metabolism to chromatin remodeling (14, 15, 16). Glutamine-dependent chromatin regulation has been implicated in innate immune memory and cell fate transitions (17, 18). In activated B cells, α-ketoglutarate-dependent H3K27me3 demethylation at the *Bcl6* locus has been shown to promote germinal center differentiation (19). However, whether mitochondrial glutaminolysis actively enhances this pathway during antigen-induced humoral immunity remains unresolved.

SIRT3 is a mitochondrial NAD^+^-dependent deacetylase that regulates oxidative metabolism, fatty acid oxidation and glutamine-linked metabolic enzymes (20, 21, 22). SIRT3 activity can be restrained by SUMOylation and enhanced by SENP1-dependent deSUMOylation, a regulatory axis previously linked to macrophage polarization and T cell memory (23, 24, 25). Its role in B cell immunity is unknown.

Here we use SIRT3 K223R knock-in mice, in which lysine 223 is replaced by arginine to prevent inhibitory SUMOylation, to test whether enhanced mitochondrial SIRT3 activity modulates germinal center B cell responses. We find that SIRT3 activation is associated with routing of glutamine-derived carbon toward α-ketoglutarate accumulation, reduced H3K27me3 enrichment at *Bcl6* regulatory regions, and reinforcement of GC-associated BCL6 and AID expression. This mechanism connects mitochondrial glutamine metabolism to adaptive immune quality control while suggesting how a pathway that improves antibody affinity can also increase autoimmune germinal center liability when chronically activated.

## Results

### SIRT3 activation intrinsically enhances germinal center responses

To determine whether mitochondrial SIRT3 regulates humoral immunity, we analyzed SIRT3 K223R knock-in mice. The mice were viable and showed normal B cell development in the bone marrow, with comparable pro-B/pre-B, immature B and mature B compartments. Splenic architecture and major immune cell frequencies were also largely preserved, indicating that constitutive SIRT3 activation does not grossly disrupt basal lymphoid development (Fig. S1). Peripheral B cell subset analysis revealed a modest reduction in marginal zone B cells and a corresponding increase in follicular B cells (Fig. 1*A*), suggesting a shift toward a compartment competent to participate in germinal center reactions.

**Figure 1:**
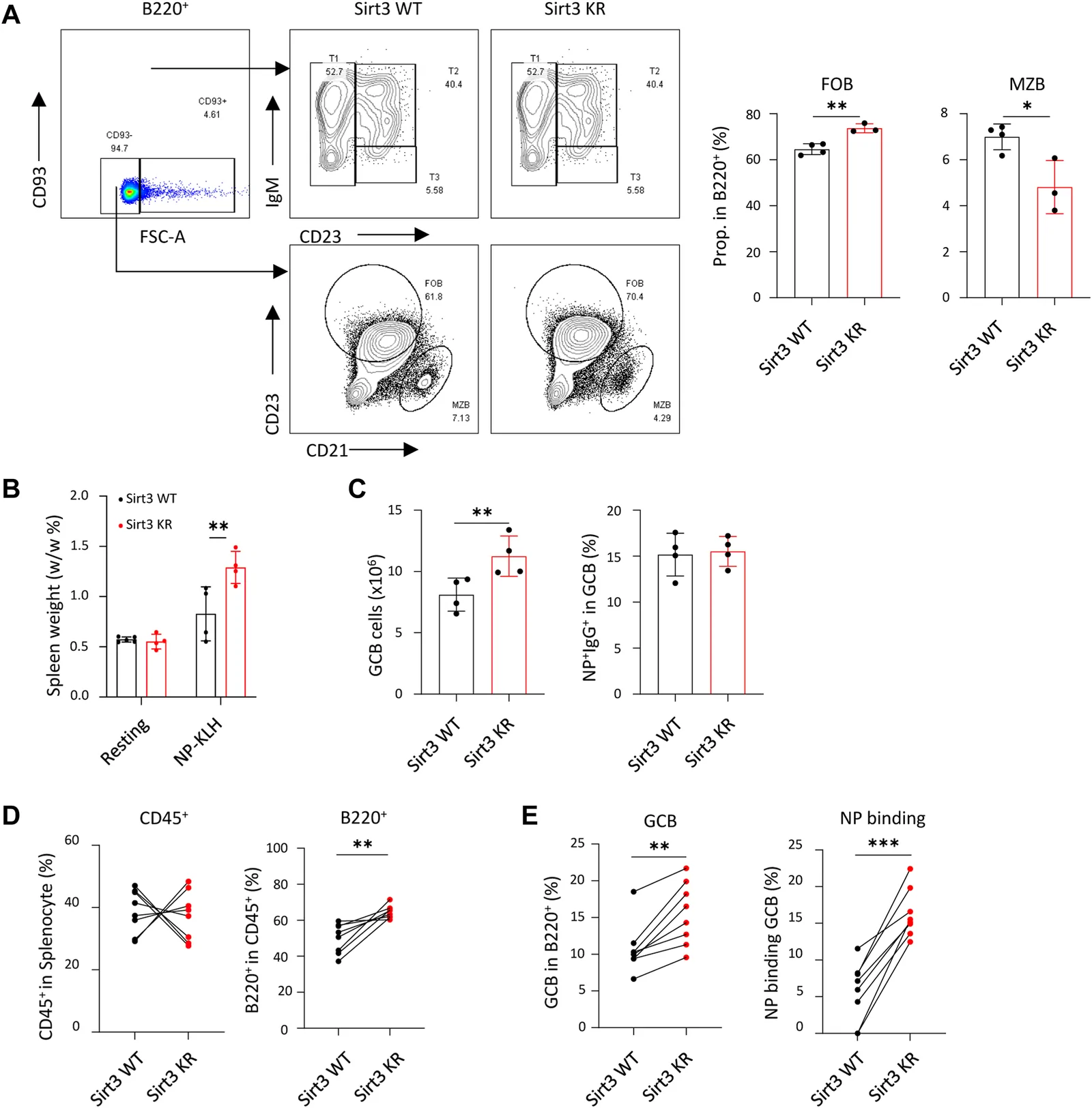
**Enhanced SIRT3 activity intrinsically enhances antigen-specific germinal center B-cell responses.***A*, flow cytometric analysis of transitional and mature splenic B-cell subsets in SIRT3 wild-type and SIRT3 K223R mice. *B*, spleen weight 14 days after NP28-KLH immunization. *C*, quantification of splenic germinal center B cells (B220^+^ GL7^+^ CD38^lo^) and Isotype-switched NP-binding cells (B220^+^ GL7^+^ CD38^lo^ IgG^+^ NP^+^) among germinal center B cells after immunization. *D*, donor contribution to total CD45^+^ hematopoietic cells and to B220^+^ B cells within the CD45^+^ compartment in competitive bone marrow chimeras. *E*, donor contribution to germinal center B cells and NP-binding germinal center B (B220^+^ GL7^+^ CD38^lo^ NP^+^) cells in competitive chimeras after immunization. Data are mean ± SD. ∗*p* < 0.05; ∗∗*p* < 0.01; ∗∗∗*p* < 0.001. Two-tailed unpaired Student’s t tests were used for *A* and *C*; two-way ANOVA was used for *B*; two-tailed paired Student’s t tests were used for *D* and *E*. n = 4 wild-type and 3 SIRT3 K223R mice in *A*; n = 4 mice per group in *B* and *C*; n = 8 chimeric mice in *D* and *E*. Data are representative of three independent experiments for *A–C* and Two independent experiments for *D* and *E*.

After NP28-KLH immunization, SIRT3 K223R mice developed larger spleens and expanded germinal center structures compared with wild-type controls (Figs. 1*B* and S2). Flow cytometry confirmed increased frequencies and absolute numbers of B220^+^GL7^+^CD38^lo^ germinal center (GC) B cells. The proportion of NP28-binding cells within the isotype-switched GCB population (B220^+^GL7^+^CD38^lo^) remained unchanged (Figs. 1*C* and S2*C*). T follicular helper cell frequencies were not increased (Fig. S3*A*), arguing that enhanced germinal center output was not secondary to augmented T cell help.

To test B cell autonomy, irradiated recipients were reconstituted with equal mixtures of CD45.1^+^ wild-type and CD45.2^+^ SIRT3 K223R bone marrow. Following NP28-KLH immunization, SIRT3 K223R-derived cells showed increased contribution to the B220^+^ B-cell compartment, GCB compartment and NP28-binding GCB cells, whereas contribution to the total CD45^+^ hematopoietic compartment was comparable (Figs. 1, *D* and *E* and S3*B*). Thus, enhanced SIRT3 activity confers a B cell-intrinsic competitive advantage during antigen-induced germinal center responses.

### SIRT3 promotes GCB cell proliferation and qualitative affinity maturation

We next asked whether SIRT3 activation enhances B cell differentiation under defined stimulation conditions. Splenic B cells activated with anti-CD40 and IL-4 generated more induced germinal center-like B cells when derived from SIRT3 K223R mice than from wild-type controls (Fig. 2*A*). SIRT3 K223R B cells also showed increased IgG1 class switching, stronger CellTrace dilution, higher BrdU incorporation and greater cell expansion during early activation (Fig. 2, *B*–*E*). These data indicate that enhanced SIRT3 activity supports both proliferation and germinal center-like differentiation in B cells.

**Figure 2:**
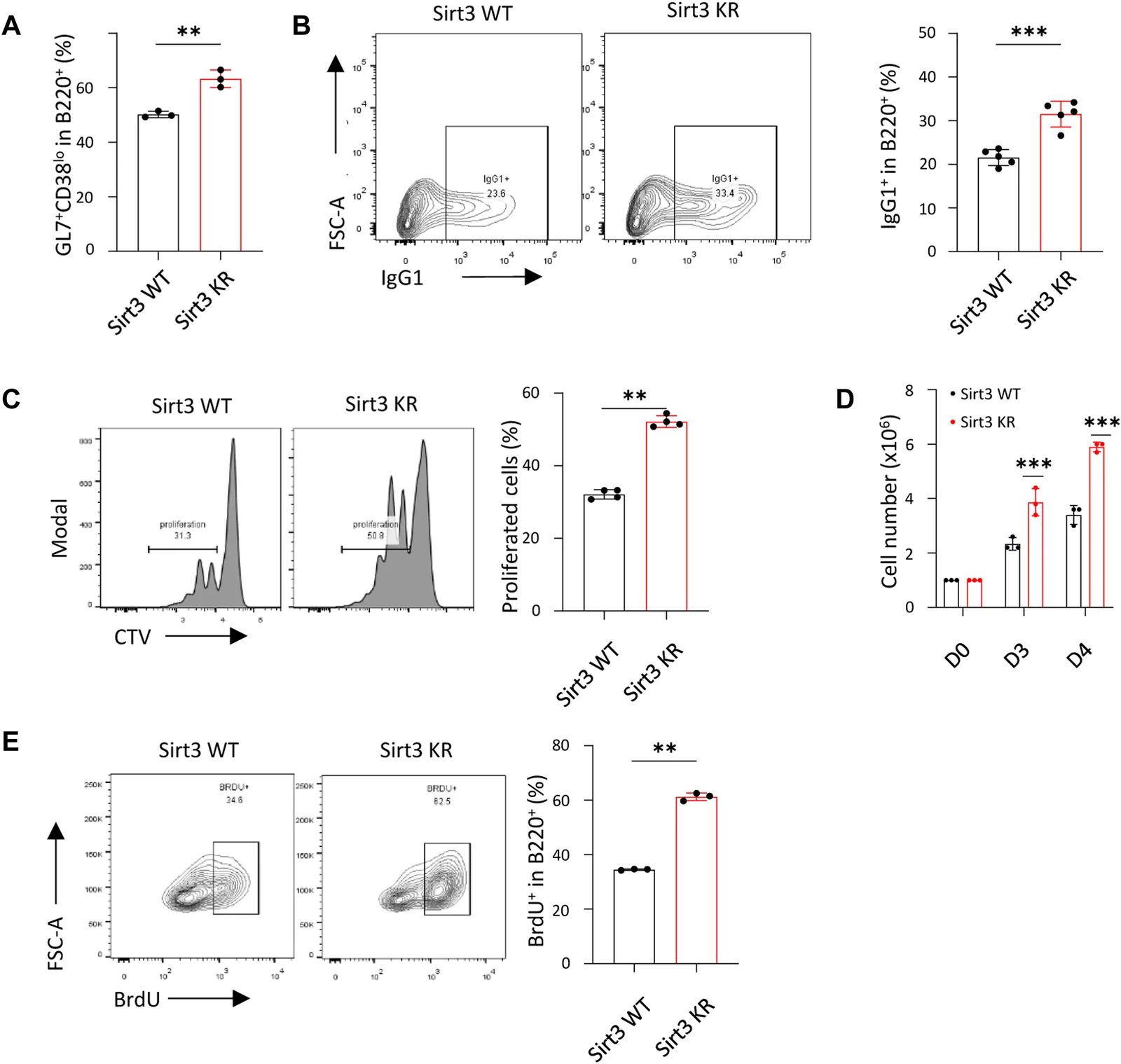
**SIRT3 activation promotes germinal center-like B cell differentiation and proliferation *in vitro*.***A*, induced germinal center-like B cells generated from anti-CD40/IL-4-activated splenic B cells. *B*, IgG1^+^ B cell differentiation. *C*, CellTrace dilution and proliferating cell quantification. *D*, cell expansion during activation. *E*, BrdU incorporation in B cells. Data are mean ± SD. ∗*p* < 0.05; ∗∗*p* < 0.01; ∗∗∗*p* < 0.001. Two-tailed unpaired Student's t tests were used. n = 3 biological replicates in *A*, *D* and *E*; n = 5 biological replicates in B; n = 4 biological replicates in *C*. Data are representative of three independent experiments for *A–D* and two independent experiments for *E*.

Consistent with enhanced germinal center output, unimmunized SIRT3 K223R mice showed increased serum IgG and IgA, whereas IgM was unchanged (Fig. 3*A*). After NP28-KLH immunization, SIRT3 K223R mice generated higher titers of high-avidity NP2-binding IgG and an increased NP2/NP36 avidity ratio (Fig. 3, *B*–*D*). Sequencing the genomic VH1-72 (VH186.2) region from sorted germinal center B cells did not reveal a global increase in mutation frequency (Fig. 3, *E* and *F*), indicating that SIRT3 activation does not simply increase activation-induced cytidine deaminase mutational burden. Instead, mutation pattern analysis showed enrichment of the affinity-promoting W33L substitution characteristic of the NP/VH186.2 response (9, 26) (Fig. 3, *E* and *G*). SIRT3 activation therefore improves the quality of affinity maturation, most likely by favoring selection or persistence of higher affinity germinal center clones rather than by increasing overall mutagenesis.

**Figure 3:**
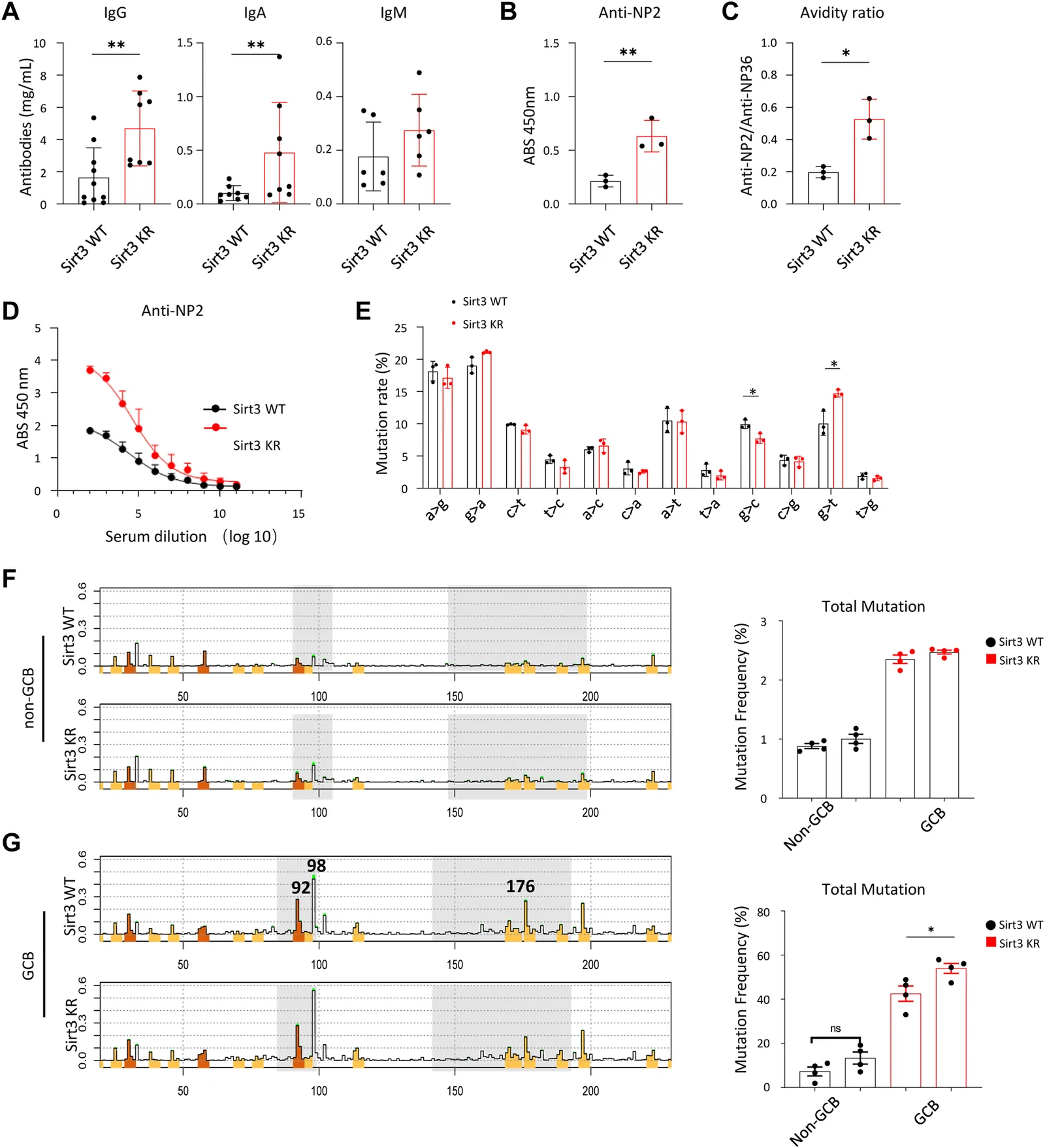
**Enhanced SIRT3 activity promotes qualitative antibody affinity maturation.***A*, basal serum immunoglobulin levels in unimmunized mice. *B–D*, Anti-NP2 IgG titers, NP2/NP36 avidity ratio and binding curves after NP28-KLH immunization. *E–G*, VH1-72 mutation pattern analysis, total mutation frequency and W33L mutation frequency in sorted GC and non-GCB populations. Data are mean ± SD. ∗*p* < 0.05; ∗∗*p* < 0.01; ∗∗∗*p* < 0.001. Two-tailed unpaired Student’s t tests were used for antibody measurements. Mutation-pattern and mutation-frequency analyses used ANOVA with multiple-comparison correction as indicated in the source data. Data are representative of two independent experiments. For *E*, the numbers of independent VH1-72 sequences analyzed were as follows: WT, n = 156, 156, 150 for three individual samples; KR, n = 156, 154, 236 for three individual samples. For *F* and *G*, DNA samples from four individual mice per group were analyzed by sequencing performed on an Illumina MiSeq instrument. Data are representative of two independent experiments for *A–D*.

### SIRT3 activation promotes an αKG-associated demethylation-permissive chromatin state

Because SIRT3 regulates mitochondrial enzymes, we asked whether its effect on germinal center B cells involves metabolic intermediates that influence chromatin. In vitro-activated SIRT3 K223R B cells expressed higher *Bcl6* and *Aicda* mRNA and protein than wild-type cells (Fig. 4, *A* and *B*). Immunoblotting showed reduced global H3K27me3 and increased histone acetylation marks in SIRT3 K223R B cells (Fig. 4*C*), consistent with a chromatin state permissive for germinal center gene expression.

**Figure 4:**
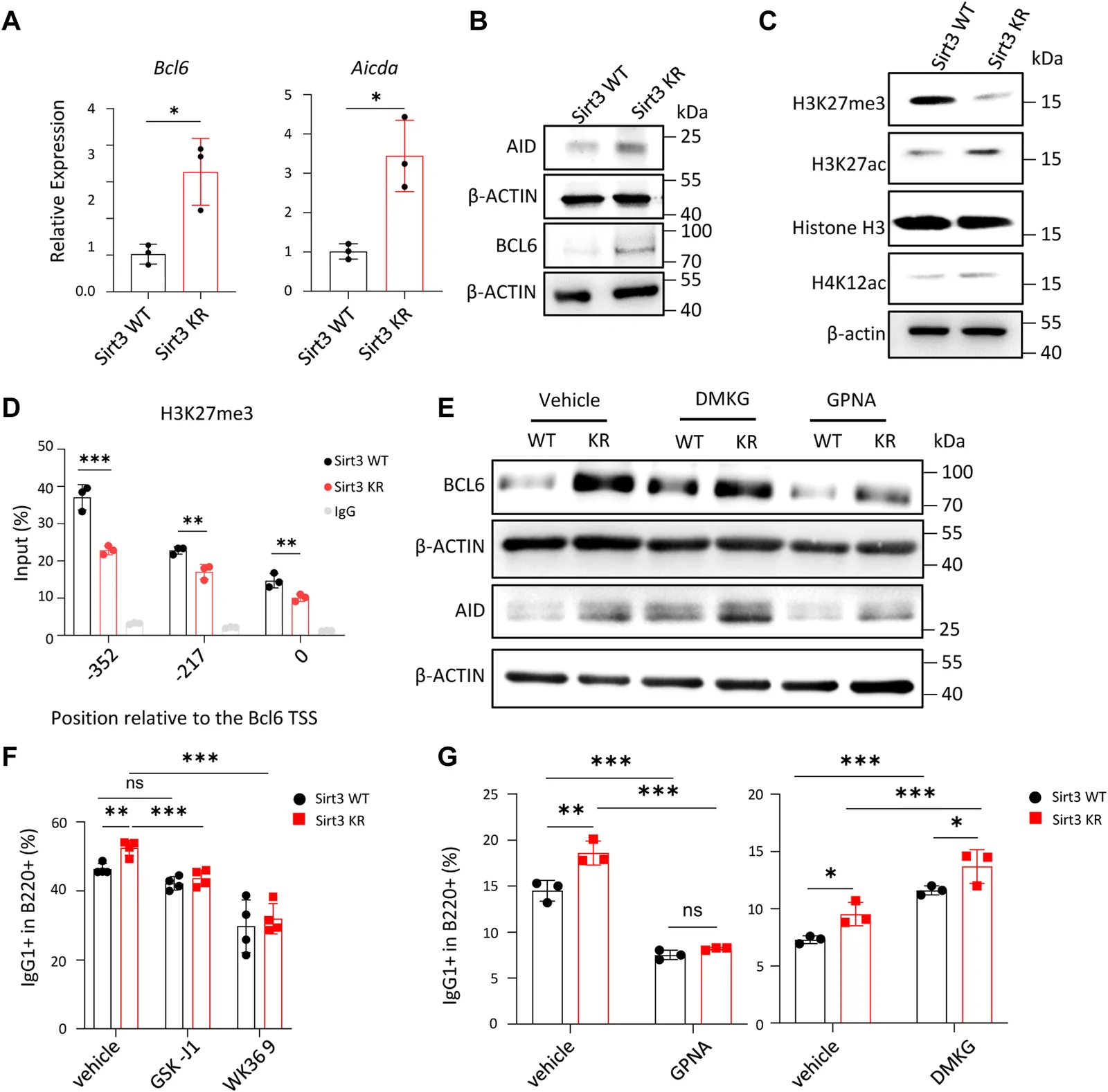
**Enhanced SIRT3 activity supports α-ketoglutarate-associated chromatin modulation at tested germinal center regulatory regions.***A* and *B*, *Bcl6* and *Aicda* mRNA and BCL6 and AID protein expression in activated B cells. *C*, global histone modification analysis. *D*, H3K27me3 enrichment at tested *Bcl6* promoter and enhancer regions; matched IgG isotype controls are shown to define nonspecific background. *E*, BCL6 and AID expression after GPNA or α-ketoglutarate treatment. *F*, IgG1 differentiation after histone demethylase or BCL6 inhibition. *G*, IgG1 differentiation after GPNA or α-ketoglutarate treatment. Data are mean ± SD. ∗*p* < 0.05; ∗∗*p* < 0.01; ∗∗∗*p* < 0.001. Two-tailed unpaired Student’s *t* tests were used for *A*; two-way ANOVA was used for *D–G* unless otherwise indicated. n = 3 or four biological replicates. Data are representative of three independent experiments.

Chromatin immunoprecipitation revealed reduced H3K27me3 enrichment at tested *Bcl6* promoter and enhancer regions in SIRT3 K223R B cells (Fig. 4*D*), consistent with prior evidence that α-ketoglutarate-dependent H3K27me3 demethylation can activate the *Bcl6* locus during germinal center B cell differentiation (19). Because α-ketoglutarate is required for Jumonji-domain histone demethylase activity, we tested whether this metabolite could phenocopy SIRT3 activation. Cell-permeable dimethyl-α-ketoglutarate increased BCL6 and AID protein expression and enhanced IgG1 differentiation in wild-type B cells (Figs. 4, *E* and *G* and S4*G*). In contrast, inhibition of glutamine metabolism by the glutamine transporter inhibitor GPNA reduced BCL6 and AID protein expression and impaired IgG1 differentiation (Fig. 4, *E* and *G*). Conversely, inhibition of KDM6A/KDM6B with GSK-J1 or inhibition of BCL6 with WK-369 impaired IgG1 differentiation (Figs. 4*F* and S4*G*). Moreover, inhibitor treatments did not affect cell viability (Fig. S6). These results support a model in which enhanced SIRT3 activity is associated with αKG-dependent reduction of H3K27me3 at the tested *Bcl6* regulatory regions.

### SIRT3 activation increases glutamine-derived αKG without a uniform increase in downstream TCA-cycle labeling

Metabolomic profiling revealed increased intracellular glutamine, glutamate, and α-ketoglutarate in activated SIRT3 K223R B cells (Figs. 5*A* and S5*B*). Seahorse analysis showed increased maximal respiratory capacity, whereas ATP levels were not increased (Figs. S4*C* and S5*A*). These findings suggest that enhanced mitochondrial capacity was not primarily directed toward increased steady-state ATP abundance.

**Figure 5:**
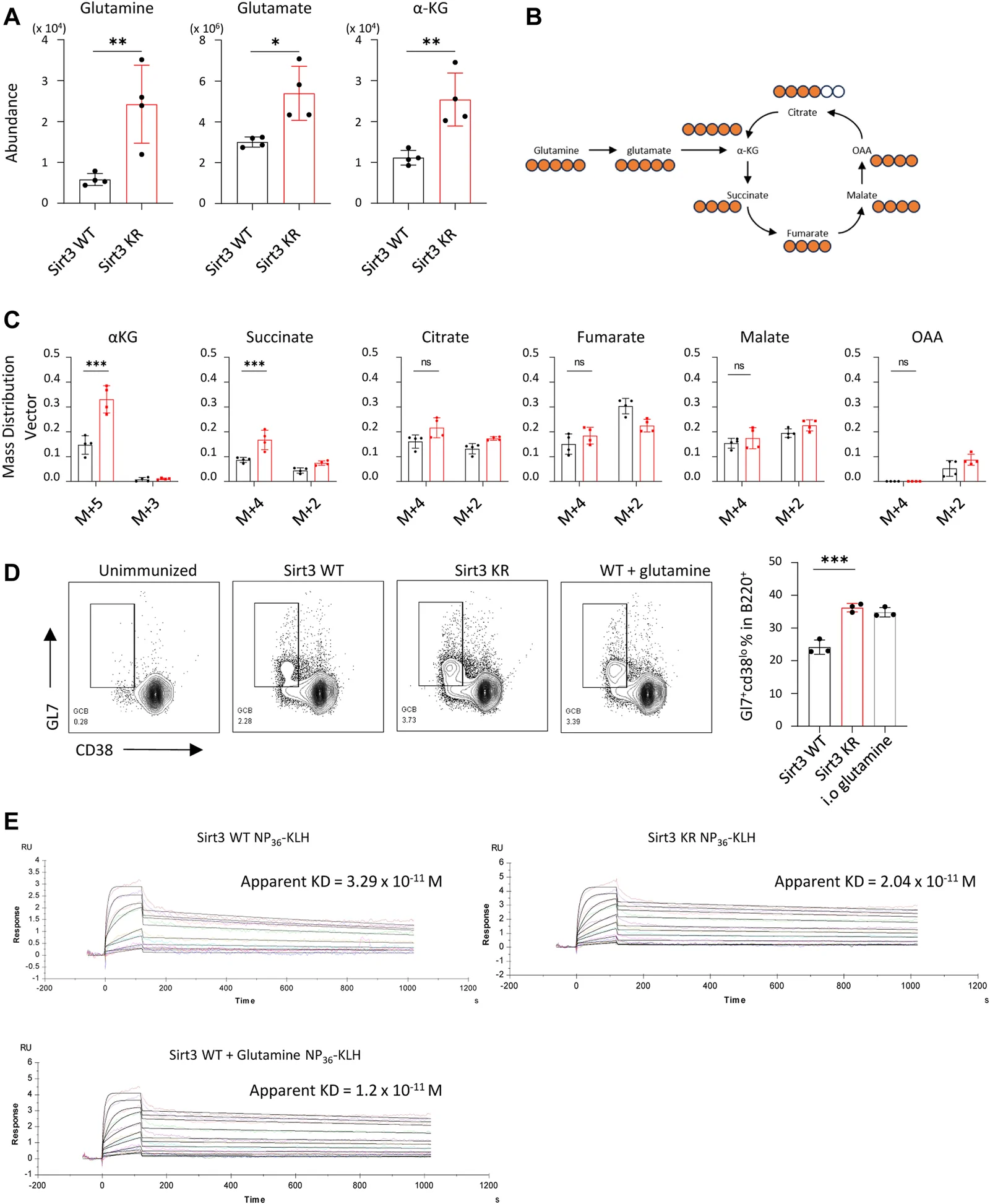
**Enhanced SIRT3 activity increases glutamine-derived α-ketoglutarate, and glutamine availability enhances germinal center output.***A*, intracellular glutamine, glutamate, and α-ketoglutarate levels in activated B cells. *B* and *C*, ^13^C_5_-glutamine tracing into α-ketoglutarate and downstream tricarboxylic acid-cycle intermediates. *D*, representative contour plots and quantification of germinal center B-cell frequencies after NP28-KLH immunization with or without glutamine supplementation. *E*, representative surface plasmon resonance sensorgrams of serum antibody binding. *Panel E* is descriptive, and no inferential group comparison was performed. Data are mean ± SD. ∗*p* < 0.05; ∗∗*p* < 0.01; ∗∗∗*p* < 0.001. Two-tailed unpaired Student’s t tests were used for *A*; two-way ANOVA was used for (*C*); one-way ANOVA with Tukey’s multiple-comparisons test was used for *D*. n = 4 biological replicates in *A* and *C*; n = 3 mice per group in (*D*).

To determine how SIRT3 activation redirects carbon flow, we performed stable isotope tracing with ^13^C_5_-glutamine. SIRT3 K223R B cells showed increased M + 5 α-ketoglutarate enrichment (Fig. 5, *B* and *C*). Isotopologue-specific analysis showed increased M + 4 succinate, whereas M + 4 malate, M + 4 citrate, M + 4 fumarate, and M + 4 OAA did not show consistent increases across the measured isotopologues (Fig. 5, *B* and *C*). These data argue against a broad, uniform increase in downstream TCA-cycle labeling. Instead, enhanced SIRT3 activity was associated with preferential accumulation of glutamine-derived α-ketoglutarate together with selective changes in downstream intermediates. To evaluate a candidate metabolic node linking SIRT3 activity to αKG production, we examined GLUD1 acetylation by co-immunoprecipitation in activated SIRT3 WT and K223R B cells. A representative immunoblot showed a lower acetylated-GLUD1 signal in K223R cells relative to WT, with comparable total GLUD1 levels (Fig. S4*F*). This direction is consistent with enhanced SIRT3 deacetylase activity; however, the experiment does not identify the responsible acetyl-lysine site, establish direct enzymatic deacetylation, or measure GLUD1 activity. We therefore consider GLUD1 a candidate SIRT3-regulated metabolic node linking glutamate-to-αKG metabolism to downstream chromatin regulation.

GPNA treatment, used here to pharmacologically restrict glutamine transport, attenuated the enhanced differentiation of SIRT3 K223R B cells, whereas α-ketoglutarate supplementation restored key features of the response (Figs. 4*G* and S4*D*). *In vivo*, glutamine supplementation in drinking water increased germinal center B-cell frequencies and was associated with an apparent improvement in representative serum-antibody SPR profiles in the NP28-KLH model, partially phenocopying the SIRT3 K223R phenotype (Fig. 5, *D* and *E*). These results indicate that glutamine availability modulates germinal center output within the SIRT3–α-ketoglutarate-associated response.

## Discussion

These findings identify enhanced SIRT3 activity as a positive modulator of glutamine-derived α-ketoglutarate accumulation and germinal center-associated chromatin regulation. Rather than broadly increasing steady-state mitochondrial energy output, SIRT3 activation increased glutamine-derived αKG and was associated with reduced H3K27me3 at tested Bcl6 regulatory regions, higher BCL6 and AID expression, and enhanced GCB proliferation and affinity maturation. The central working model supported by the current data is a SIRT3-activity-linked glutamine-αKG-chromatin axis. A representative co-immunoprecipitation experiment showed lower GLUD1 acetylation in K223R B cells, nominating GLUD1 as a candidate metabolic node; however, site-specific substrate mapping, quantitative validation, and enzymatic activity assays are required before concluding direct catalytic regulation.

This model extends current views of immune metabolism in two ways. First, mitochondrial metabolism in germinal center B cells may provide metabolite-linked epigenetic modulation without a uniform increase in downstream tricarboxylic acid-cycle labeling, extending previous links between B-cell metabolism, *Bcl6* locus remodeling, and affinity selection (9, 19). Second, nutrient availability is linked to qualitative immune output: glutamine supplementation enhances germinal center responses and is associated with an apparent improvement in representative SPR profiles, whereas interference with glutamine transport or histone demethylase activity blunts germinal center-like differentiation.

Enhanced germinal center identity may, however, carry a tolerance cost because dysregulated germinal center and extrafollicular B-cell responses contribute to autoantibody production in systemic autoimmunity (27). In a pristane-induced lupus model (28, 29), SIRT3 K223R mice showed increased splenic germinal center B-cell frequencies 6 months after pristane administration, accompanied by higher anti-nuclear antibody titers and greater renal IgG deposition than wild-type controls (Fig. S5, *C*–*F*). Thus, chronic activation of the SIRT3–α-ketoglutarate-associated pathway was linked to greater autoreactive germinal center expansion in this model.

Several questions remain. Our gain-of-function data show that enhanced SIRT3 activity potentiates; Whether SIRT3 serves as an indispensable physiological mediator in wild-type GCB cells will require future investigation using B cell-specific SIRT3-deficient models. Genome-wide chromatin profiling will be required to determine whether SIRT3-α-ketoglutarate signaling affects a broad germinal center regulatory network or acts most strongly at key loci such as *Bcl6*. In addition, GLUD1 substrate mapping, quantitative acetylation analysis, and enzymatic activity measurements will be needed to determine the biochemical consequence and causality of GLUD1 deacetylation. Overall, the present data support a focused model in which enhanced SIRT3 activity promotes germinal center epigenetic modulation and influences the quality and autoimmune liability of adaptive humoral immunity.

## Experimental procedures

### Mice

All experiments utilized mice on a C57BL/6 background. SIRT3 K223R knock-in mice were generated *via* CRISPR-Cas9 by Shanghai Bangyao Biotechnology Co., Ltd. Both SIRT3 WT and KR mice were derived from F1 homozygous offspring of heterozygous SIRT3 WT/SIRT3 KR breeders and maintained for over 20 generations. Male mice aged 8 to 12 weeks (body weight 20–25 g) were used for immunization, and 6-week-old mice were used as bone marrow donors. CD45.1 congenic mice (B6.SJL-Ptprc^a^ Pepc^b^/BoyJ, originally from the Jackson Laboratory) were maintained in-house. Recipient mice (CD45.1/CD45.2 heterozygotes) for bone marrow chimeras were F1 offspring generated by crossing homozygous CD45.2 and CD45.1 parent mice. All mice were housed under specific pathogen-free (SPF) conditions at 22 ± 2 °C with 45 to 65% humidity and a 12-h light/12-h dark cycle and had free access to irradiated standard laboratory chow and autoclaved water. Mice were allowed to acclimate for at least 1 week before any experimental procedure. All animal procedures were approved by the Shanghai Jiao Tong University School of Medicine IACUC (Approval #A-2022-093). Euthanasia was performed by CO_2_ inhalation followed by cervical dislocation. Genotyping was performed using tail or toe DNA and a genomic DNA extraction kit (Vazyme), followed by PCR amplification and Sanger sequencing.

Non-randomized group allocation and non-blinded procedures were used. Group sizes were derived from pilot data and our prior work. Animals meeting prespecified exclusion criteria were removed from analysis.

### Cell culture and treatments

Primary splenic B cells were purified from resting mice aged 8 to 12 weeks using negative selection magnetic beads (STEMCELL Technologies). Cells were cultured at 1 × 10^6^ cells/ml in RPMI 1640 medium (Sigma-Aldrich) supplemented with 10% (v/v) heat-inactivated FBS (ExpCell), 1% penicillin/streptomycin (Gibco; 100 U/ml penicillin and 100 μg/ml streptomycin), 1% GlutaMAX (Gibco) or 2 mM L-glutamine (Gibco), 1% sodium pyruvate (Gibco), 1% MEM non-essential amino acids (Gibco), and 55 μM 2-mercaptoethanol (Gibco). Cells were maintained at 37 °C in a humidified incubator with 5% CO_2_.

For *in vitro* activation, cells were stimulated with 2 μg/ml anti-CD40 (Mabtech) and 40 ng/ml IL-4 (Invitrogen) for 4 days. Concurrently, cells were treated with vehicle (DMSO) or the following inhibitors (all from MCE): 10 μM GPNA (glutamine transporter inhibitor), 10 μM GSK-J1 (KDM6A/KDM6B histone demethylase inhibitor), or 10 μM WK-369 (BCL6 inhibitor). To assess the metabolic-epigenetic interplay, 5 mM cell-permeable dimethyl-αKG (Sigma-Aldrich) was administered. All treatments were maintained for 4 days.

For BrdU incorporation, 10 μM BrdU (Sigma-Aldrich) was added to the culture medium for the last 24 h. Cells were then harvested, fixed, permeabilized, and stained with an anti-BrdU antibody followed by flow-cytometric analysis.

### ^13^C_5_-glutamine tracing and metabolic flux analysis

Primary splenic B cells were first activated with 2 μg/ml anti-CD40 and 40 ng/ml IL-4 in complete RPMI 1640 medium for 4 days as described above. Activated B cells were then washed twice and resuspended at 1 × 10^6^ cells/ml in glutamine-free RPMI 1640 medium (Shanghai BasalMedia) supplemented with 1% penicillin/streptomycin, 1% sodium pyruvate, 1% MEM non-essential amino acids, 55 μM 2-mercaptoethanol, 2 μg/ml anti-CD40, 40 ng/ml IL-4, and 2 mM ^13^C_5_-L-glutamine (MCE), without FBS. Cells were cultured for 24 h at 37 °C in a humidified 5% CO_2_ incubator. In parallel, an unlabeled control culture was identically set up but with 2 mM unlabeled L-glutamine instead, to provide natural isotope abundance backgrounds.

After incubation, cells were harvested, washed twice with ice-cold PBS, and immediately quenched by adding 400 μl of ice-cold 80% (v/v) methanol per sample. Samples were ultrasonicated and centrifuged at 18,000*g* for 15 min at 4 °C. The supernatant was split into two equal portions and evaporated to dryness by centrifugal concentration. One portion was reconstituted in 100 μl of 80% methanol for analysis of glutamine-derived metabolites; the other was reconstituted in 100 μl of deionized water for CoA detection.

Metabolite analysis was performed by Metabo-Profile Biotechnology (Shanghai) Co, Ltd using a Waters ACQUITY I-Class UPLC system coupled to a Xevo TQ-S tandem quadrupole mass spectrometer (Waters). The source parameters were set as follows: capillary voltage 2.5 kV for glutamine metabolites (ESI^-^) and 3.0 kV for CoA (ESI^+^); source temperature 150 °C; desolvation temperature 500 °C; desolvation gas flow 1000 L/h.

For glutamine-derived metabolites, separation was achieved on an ACQUITY UPLC BEH HILIC column (2.1 × 100 mm, 1.7 μm; Waters) maintained at 40 °C. The mobile phase consisted of A: 5% acetonitrile in water containing 20 mM ammonium hydroxide and 20 mM ammonium acetate (without further pH adjustment) and B: acetonitrile. The gradient program was: 0 to 3.5 min, 85%→32% B; 3.5 to 12 min, 32%→2% B; 12 to 16.5 min, 2% B; 16.5 to 17 min, 2%→85% B; 17 to 25.5 min, 85% B, at a flow rate of 0.4 ml/min. For CoA species, separation was carried out on an ACQUITY UPLC BEH C18 column (2.1 × 100 mm, 1.7 μm; Waters) maintained at 30 °C. The mobile phase consisted of A: water containing 10 mM ammonium acetate and 0.05% ammonium hydroxide (v/v) and B: 95% acetonitrile/water containing 10 mM ammonium acetate and 0.05% ammonium hydroxide (v/v). The gradient was: 0 to 1.2 min, 3% B; 1.2 to 1.7 min, 3%→11% B; 1.7 to 7 min, 11%→13% B; 7 to 7.2 min, 13%→20% B; 7.2 to 9.4 min, 20%→100% B; 9.4 to 11.4 min, 100% B; 11.4 to 11.7 min, 100%→3% B; 11.7 to 13 min, 3% B, at a flow rate of 0.2 ml/min. The injection volume was 5 μl for both methods. Solvent blanks were injected every 6 to 10 samples, and the injection order was randomized. Data were acquired and processed with MassLynx v4.1 software.

For isotope tracing, isotopologue mass distribution vectors (MDVs) were calculated as the fractional abundance of each isotopologue relative to the sum of all isotopologues for a given metabolite. Natural ^13^C and ^15^N abundance contributions were corrected by subtracting the isotopologue fractions measured in the unlabeled control samples analyzed in the same batch. Corrected MDVs were normalized to cell number, and results are presented as MDVs to compare pathway activities. All tracing experiments were performed with four independent biological replicates, and data are shown as means ± SD.

### Immunizations and disease models

For T-dependent responses, mice were immunized intraperitoneally (i.p.) with 10 mg/kg NP_28_-KLH (Biosearch Technologies) emulsified in MnJ(β) adjuvant (Mnstarterbio) in a 200 μl volume. Serum was collected from the tail vein on days 7, and NP-specific antibody titers were measured by ELISA For germinal center analysis. For germinal center analysis, mice were euthanized by CO_2_ inhalation followed by cervical dislocation on day 14, and spleens were harvested for flow cytometry.

For bone marrow chimeras, CD45.1/CD45.2 heterozygous recipient mice (8–12 weeks old) were lethally irradiated with a single dose of 8 Gy (X-ray). Bone marrow cells were isolated from femurs and tibias of CD45.1 SIRT3 WT and CD45.2 SIRT3 KR donor mice. After red blood cell lysis, 2 × 10^6^ cells from each donor type were mixed at a 1:1 ratio and injected intravenously into each recipient *via* the tail vein. Chimeric mice were maintained on autoclaved water containing antibiotics (neomycin and polymyxin B) for 4 weeks. After a total reconstitution period of 12 weeks, peripheral blood chimerism was confirmed by flow cytometry (CD45.1 *versus* CD45.2). Mice were then immunized i.p. with 10 mg/kg NP_28_-KLH in MnJ(β) adjuvant and analyzed on day 14 as above.

Lupus was induced in 3-month-old SIRT3 WT and KR male mice by a single i.p. injection of 500 μl pristane (Merck,). Control mice received an equal volume of sterile PBS. Mice were euthanized 6 months post-injection, and tissues were harvested.

For *in vivo* glutamine supplementation, L-glutamine was dissolved in drinking water (referred to as I.O. in figures) at a final concentration of 0.2 mM and provided ad libitum to mice. The treatment was continued until the day of sacrifice. Fresh glutamine-containing water was replaced every 2 to 3 days. Control animals received regular drinking water without glutamine.

### Flow cytometry

Single-cell suspensions from spleen and bone marrow were prepared by mechanical dissociation. Red blood cells were lysed, and cells were stained with Zombie Violet (Thermo Fisher Scientific, 5 μM, 4.5 min in 37 °C). For surface staining, 2 × 10^6^ cells were blocked with anti-CD16/32 (BioLegend,) and stained with fluorochrome-conjugated antibodies for 15 min at room temperature. For intracellular staining, cells were fixed and permeabilized (Invitrogen) before antibody incubation. Mitochondrial probes (MitoSOX, MitoTracker, TMRM) were stained in culture medium at 37°C for 15 to 30 min.

Data were acquired on a BD FACSAria II flow cytometer. Compensation was performed using single-stained cells or fluorescence minus one (FMO) controls. Dead cells and doublets were excluded from the analysis by gating on Zombie Violet-negative events and FSC-A/FSC-H properties. All antibodies and their dilutions are listed in Table S1. Data were analyzed using FlowJo software (version 10).

### Immunoblotting

Cells were washed twice with ice-cold PBS and lysed in RIPA buffer supplemented with protease and phosphatase inhibitor cocktails on ice for 30 min with intermittent vortexing. Lysates were centrifuged at 12,000*g* for 5 min at 4 °C, and the supernatants were collected. Protein concentrations were determined using a BCA protein assay kit (Yeasen# 20200ES76) according to the manufacturer’s instructions. Equal amounts of protein 10 μg per lane were mixed with loading buffer, boiled at 95 °C for 10 min, and separated by SDS-PAGE on 12% polyacrylamide gels alongside a prestained protein ladder (Vazyme# MP102–01). Electrophoresis was performed in Tris-glycine-SDS running buffer at 80 V for 20 min followed by 120 V for 1 h.

Proteins were transferred onto PVDF membranes (0.45 μm pore size) in Tris-glycine transfer buffer (25 mM Tris, 192 mM glycine, 10% methanol) at 100 V for 90 min at 4 °C. After transfer, membranes were blocked with 5% non-fat dry milk in TBST (Tris-buffered saline with 0.1% Tween-20) for 1 h at room temperature.

Membranes were incubated overnight at 4 °C with primary antibodies diluted in TBST containing 3% BSA (w/v). After three washes with TBST (10 min each), membranes were incubated with the appropriate HRP-conjugated secondary antibodies diluted in TBST-BSA for 40 min at room temperature. Following additional washing, protein bands were visualized using enhanced chemiluminescence (ECL) substrate (Invitrogen). Images were acquired on a Tanon 2500B chemiluminescence imaging system and quantified using the associated software. For normalization, β-actin was used as the loading control for total protein levels; for histone modifications, Histone H3 served as the internal control. All primary and secondary antibodies are listed in Table S1. Each immunoblot was repeated at least three times with independently prepared samples.

### Co-immunoprecipitation

Cell lysates were prepared from in vitro-activated B cells isolated from Sirt3 WT and Sirt3 K223R mice. Total acetylated proteins were immunoprecipitated using an anti-acetyl-lysine antibody, and the immunoprecipitates were subjected to immunoblot analysis with an anti-GLUD1 antibody to detect acetylated GLUD1. Total GLUD1 protein levels were measured in input lysates. Acetylated GLUD1 signal was assessed relative to total GLUD1.

#### Seahorse assay

Mitochondrial function was assessed by measuring the oxygen consumption rate (OCR) of B cells using an Agilent Seahorse XF Analyzer according to the manufacturer's instructions for the Mito Stress Test. On the day prior to the assay, a Seahorse XF96 cell culture microplate was coated with Cell-Tak (Corning). The Cell-Tak was diluted to a final concentration of 22.4 μg/ml in 0.1 M sodium bicarbonate, and 25 μl of this solution was added to each well and incubated for 20 min at room temperature. The wells were then washed twice with 200 μl of sterile water. B cells were seeded at a density of 2 × 10^4^ cells per well in the coated plate. On the day of the assay, the culture medium was replaced with Seahorse XF DMEM assay medium (pH 7.4), and the plate was incubated in a non-CO_2_ incubator at 37 °C for 45 to 60 min prior to the measurement. OCR was measured under basal conditions and following the sequential injection of oligomycin (1 μM), FCCP (1 μM), and a mixture of rotenone and antimycin A (0.5 μM each). Data were acquired and analyzed using the Agilent Seahorse Wave software.

#### qPCR

Total RNA was extracted from cells using TRIzol reagent (Invitrogen# 15596018CN) according to the manufacturer's protocol. Reverse transcription was performed with the HiScript III RT SuperMix (Vazyme# R323–01) on a conventional thermal cycler under the following conditions: 37 °C for 15 min, 85 °C for 5 s, and then held at 10 °C. Quantitative PCR was carried out on a LightCycler 96 Instrument (Roche) using SYBR Green Master Mix (Vazyme# Q111–02). The thermal cycling protocol was: 95 °C for 5 min, followed by 50 cycles of 95 °C for 10 s, 60 °C for 30 s, and 72 °C for 20 s. Melting curve analysis was performed to confirm the specificity of each amplification, and standard curves for each primer pair confirmed amplification efficiencies within the range of 90%–110%. All reactions were run in triplicate. 18S rRNA was used as the endogenous control for normalization. For relative quantification, the 2^-ΔΔC^ method was applied. Briefly, the average Ct values of the target gene and the endogenous reference (18S rRNA or ACTB) from triplicate reactions were used to calculate ΔCt for each sample (ΔCt = Ct^target^ - Ct^18S^). The ΔΔCt was then obtained by subtracting the ΔCt of the Sirt3 WT from the ΔCt of Sirt3 KR. The fold change in expression was calculated as 2^-ΔΔCt^. The primer sequences used in this study are listed in Table S1.

#### Somatic hypermutation (SHM)

##### Somatic hypermutation (SHM) analysis

Genomic DNA was extracted from sorted GCB cells (B220^+^GL7^+^) and non-GCB cells (B220^+^GL7^-^). The VH186.2 (VH1-72) segment was amplified by nested PCR using primers listed in Table S1. PCR was performed with a high-fidelity DNA polymerase (Vazyme#P521). the JH4 intronic region was amplified in a separate reaction. The first-round PCR was performed with primers 5′- CATGGGATGG AGCTGTATCATGC -3′ and 5′- CTCACAAGAGTCCGATAGACCCTG -3′ for 14 cycles at 94 °C (30 s), 55 °C (30 s), and 72 °C (90 s). One microliter of the first-round product was then re-amplified with nested primers 5′- GGTGACAATGACATCCACTTTGC -3′ and 5′- GAC TGTGAGAGTGGTGCCTTG -3′ for 21 cycles at 94 °C (30 s), 55 °C (30 s), and 72 °C (30 s). Purified PCR products were cloned into a pTOPO-T vector (Vazyme# C603) and transformed into competent *E. coli* (Vazyme# C502). Individual colonies were randomly picked and sequenced by Sanger sequencing. For each of three independent biological replicates per genotype, the following numbers of independent VH1-72 sequences were obtained: WT, 156, 156, 150; KR, 156, 154, 236. Sequences were aligned to the germline VH186.2 reference using IMGT/HighV-QUEST, and the total mutation frequency (mutations per base pair, excluding the CDR3 region because of junctional diversity) was calculated for each sequence.

For high-throughput sequence alignment, the libraries were pooled and sequenced on an Illumina MiSeq platform as previously reported (30). Reads were quality-filtered, assembled, and aligned to germline VH references using MEGA and custom scripts.

### Histology and ELISA

Spleens were fixed in 4% paraformaldehyde (PFA) at 4 °C for 24 h, embedded in paraffin, and sectioned at 5 μm. Deparaffinized sections were stained with hematoxylin and eosin, dehydrated through graded ethanol, cleared in xylene, and mounted with neutral balsam. Objectives of 10× or 40× were used as appropriate. Kidneys were fixed in 4% PFA, cryoprotected in 30% sucrose, embedded in OCT compound, and cut into 8 μm cryosections. Sections were blocked with Immunostaining Blocking Buffer (Beyotime# P0102) for 1 h at room temperature, then incubated overnight at 4 °C with a primary antibody against mouse IgG. After washing, sections were incubated with fluorophore-conjugated secondary antibody for 40 min at room temperature in the dark. Nuclei were counterstained with DAPI for 5 min, and slides were mounted with Antifade Mounting Medium (Beyotime# P0126). Images were captured using a Leica DM250 fluorescence microscope equipped with an Olympus DP28 digital camera and cellSens software. Mean IgG fluorescence intensity was measured in randomly selected glomeruli across the renal cortex. To avoid bias, all glomeruli within each randomly chosen field were quantified. A total of 18 glomeruli from 3 WT mice (6, 5, and seven per mouse) and 17 glomeruli from 3 KR mice (5, 5, and seven per mouse) were analyzed. Differences were tested with a mixed-effects model treating glomeruli as observations nested within individual mice, with genotype as a fixed effect; genotype contrasts were assessed with a *t* test on estimated marginal means.

Serum NP-specific antibody titers were measured by ELISA. High-binding 96-well plates were coated with 0.01 mg/ml NP_2_-BSA or NP_36_-BSA (Biosearch Technologies) in carbonate-bicarbonate buffer (pH 9.6) overnight at 4 °C. Plates were washed with PBST and blocked with PBST containing 1% BSA for 1 h at room temperature. Serum samples were serially diluted 10-fold (starting at 1:100) in blocking buffer across ten dilutions and incubated for 2 h at room temperature. Bound immunoglobulins were detected with HRP-conjugated goat anti-mouse IgG (Fc-specific; Invitrogen# 31439) followed by TMB substrate for 30 min at room temperature. The reaction was stopped with 2 M H_2_SO_4_, and absorbance was read at 450 nm on a microplate reader. Total IgG, IgM, and IgA were quantified by ELISA using Invitrogen kits (Thermo Fisher Scientific#88–50400–22, #50246320, #50246319) according to the manufacturer’s instructions. Acetyl-CoA levels were quantified using a commercial kit (BioVision#K317-100). Serum antinuclear antibody (RNP) levels were measured with a commercial ELISA kit (Shanghai Mlbio Biotechnology#) according to the manufacturer’s protocol. Serum was diluted 1:100.

### ChIP-qPCR

For ChIP-qPCR, in vitro-activated B cells were counted, and 5 × 10^6^ cells per sample were used. Cells were fixed with 1% formaldehyde for 15 min at room temperature. Cells were lysed sequentially in cell and nuclear lysis buffers, followed by chromatin shearing by sonication. Chromatin was sheared by sonication to an average fragment size of 100 to 300 bp. Soluble chromatin fragments were immunoprecipitated with an anti-H3K27me3 antibody. Matched IgG isotype controls and input DNA were included to define nonspecific background and normalize enrichment. Beads were treated with proteinase K, DNA was extracted using a DNA extraction kit (CST), and recovered DNA was analyzed by qPCR. Recovered DNA was analyzed by qPCR using specific primers listed in Table S1.

### Surface plasmon resonance (SPR)

All SPR experiments were performed at 25 °C on a Biacore 8K system (Cytiva) with a data collection rate of 10 Hz. HBS-EP + buffer (pH 7.4, Cytiva, #BR-1006-69) was used as the running buffer. A protein A sensor chip (Cytiva, #29127556) was preconditioned, and the ligand (anti-NP antibody) was captured at a density of less than 100 RU by a 60 s injection at 10 μl/min. A reference flow cell was activated and blocked in parallel without antibody capture, and its signal was subtracted from all sensorgrams. The analyte, NP36-BSA (molecular mass 74.0 kDa), was serially diluted in running buffer to concentrations ranging from 0.0098 to 10 nM, and each concentration was injected at 50 μl/min for 120 s, followed by a 900 s dissociation phase. Blank injections of running buffer were also performed for double-referencing. The surface was regenerated between cycles with a 30 s pulse of 10 mM glycine (pH 1.5). All sensorgrams were processed, double-referenced, and globally fitted to a 1:1 Langmuir binding model using Biacore Insight Evaluation Software to derive association rate constants (ka), dissociation rate constants (kd), and equilibrium dissociation constants (KD).

### Statistical analysis

Data are presented as mean ± SD. Statistical analyses were performed using GraphPad Prism 8.0. Two-group comparisons were performed using two-tailed Student’s t tests. Multiple comparisons were analyzed using one-way or two-way ANOVA followed by Tukey’s multiple-comparisons test unless otherwise indicated. The mouse was the biological unit for *in vivo* experiments unless otherwise specified. For renal IgG quantification, glomerular measurements were analyzed as observations nested within mouse using a mixed-effects model. Sequence-derived analyses and their sample sizes are described in the Figure 3 legend. Significance was defined as ∗*p* < 0.05, ∗∗*p* < 0.01, and ∗∗∗*p* < 0.001.

## Data availability

Data supporting the findings are provided in the manuscript and supporting information. The raw mass spectrometry data and associated metadata generated in this study have been submitted to MetaboLights under provisional submission identifier MTBLS15385. The raw Illumina MiSeq sequencing data for somatic hypermutation analysis have been deposited in the NCBI Sequence Read Archive (SRA) under BioProject accession number PRJNA1515973. Additional source data and materials are available from the corresponding author, Jinke Cheng (jkcheng@shsmu.edu.cn), upon reasonable request.

## Supporting information

This article contains supporting information.

## Conflict of interest

The authors declare that they have no conflicts of interest with the contents of this article.

## Supporting information

Figure S5

Figure S6A
