# Supplementary material for: Enhanced SIRT3 activity potentiates germinal center epigenetic modulation through α-ketoglutarate
Source: J Biol Chem. 2026 Sep 12;302(10):113552. doi: 10.1016/j.jbc.2026.113552 (PMC13676980; doi:10.1016/j.jbc.2026.113552)
Supplement: Supplementary file 1 [file mmc1.pdf]

Supplementary Table 1. Reagents and tools

Category / resource	Source	Identifier or catalog number	Notes / revision status
**Mouse lines**			
SIRT3 K223R knock-in	Shanghai Bangyao Biotechnology	CRISPR-Cas9, C57BL/6, K223R mutation	
CD45.1 congenic mice	In-house breeding	CD45.1 congenic on C57BL/6	
**Immunogens and antigens**			
NP28-KLH	Biosearch Technologies	Cat# N-5060-25	
NP2-BSA / low-ratio NP-BSA	Biosearch Technologies	Cat# N-5050XL-10	Used for high-avidity anti-NP ELISA;
NP36-BSA / high-ratio NP-BSA	Biosearch Technologies	Cat# N-5050H-10	Used for total anti-NP ELISA;
**Stimuli, inhibitors and metabolites**			
Anti-CD40	Mabtech	Clone FGK45; Cat# 3625-2-500	
IL-4	Invitrogen	Cat# PMC0045	
EasySep™ Mouse Pan-B Cell Isolation Kit	STEMCELL Technologies	Cat# #19844	
RPMI 1640 Medium	Sigma-Aldrich	Cat# # R8758	
RPMI 1640 Medium	Shanghai BasalMedia	Cat# # L210KJ	
Fetal Bovine Serum (FBS)	ExpCell	Cat# # FSP500	
Penicillin/Streptomycin	Gibco	Cat# # 15140-122	
GlutaMAX™	Gibco	Cat# # 35050061	
L-Glutamine	Gibco	Cat# # 25030-081	
Sodium Pyruvate	Gibco	Cat# # 11360-070	
Non-Essential Amino Acids	Gibco	Cat# # 11140050	
GPNA	MedChemExpress	Cat# HY-W011391; 10 μM	
GSK-J1	MedChemExpress	Cat# HY-15648; 10 μM	
WK-369, BCL6 inhibitor	MedChemExpress	Cat# HY-161138; 10 μM	
dimethyl- αKG	Sigma-Aldrich	Cat# 75890; 5 mM	
$13C_5$-glutamine	MedChemExpress	Cat# HY-N0390S1; 2 mM	
Neomycin Sulfate	Gibco	Cat# 21810031	
Polymyxin B Sulfate	Gibco	Cat# 21850029	
pristane	Merck	Cat# P2870	
Cell-Tak	Corning	Cat# 354240	
**Flow cytometry reagents**			
Zombie Violet viability dye	Thermo Fisher Scientific	Cat# L34965	
Anti-CD16/32 Fc block	BioLegend	Cat# 101320	
CellTrace Violet	Invitrogen	Cat# C34571	
MitoSOX	Thermo Fisher Scientific	Cat# M36008	
MitoTracker	Thermo Fisher Scientific	Cat# M7513	
TMRM	Thermo Fisher Scientific	Cat# T668	
**Flow cytometry antibodies**	**BioLegend / BD Biosciences**	**See full panel below**	
Anti-mouse CD19	BioLegend	Cat# 115521	6D5 (Alexa Fluor® 488)
Anti-mouse GL7	BioLegend	Cat# 144603	GL7 (FITC)
Anti-mouse CD38	BioLegend	Cat# 102718	90 (PE/Cyanine7)
Anti-mouse B220	BioLegend	Cat# 103212	RA3-6B2 (APC)
Anti-mouse CD45.1	BioLegend	Cat# 110741	A20 (Brilliant Violet 510™)
Anti-mouse CD45.2	BioLegend	Cat# 109828	104 (PerCP/Cyanine5.5)
Anti-mouse IgG1	BioLegend	Cat# 406619	RMG1-1 (APC/Cyanine7)
Anti-mouse IgG	BioLegend	Cat# 405315	Poly4053 (PE/Cyanine7)
Anti-mouse CD138	BioLegend	Cat# 142506	281-2 (APC)
Anti-mouse CD21	BioLegend	Cat# 123409	7E9 (PE)
Anti-mouse CD23	BioLegend	Cat# 101629	B3B4 (APC/Cyanine7)
Anti-mouse CXCR5	BioLegend	Cat# 145503	L138D7 (Biotin)
Anti-mouse PD-1	BioLegend	Cat# 135209	29F.1A12 (APC)
Anti-mouse BrdU	BioLegend	Cat# 364109	3D4 (PerCP/Cyanine5.5)
Anti-mouse CD86	BioLegend	Cat# 105007	GL-1 (PE)
Anti-mouse IgD	BioLegend	Cat# 405715	11-26c.2a (APC/Cyanine7)
Anti-mouse IgM	BioLegend	Cat# 406507	RMM-1 (PE)
Anti-mouse CD4	BioLegend	Cat# 100422	GK1.5 (PE/Cyanine7)
Anti-mouse CD8	BioLegend	Cat# 100714	53-6.7 (APC/Cyanine7)
Anti-mouse CD11b	BioLegend	Cat# 101215	M1/70 (PE/Cyanine7)
Anti-mouse F4/80	BioLegend	Cat# 123135	BM8 (Brilliant Violet 510™)
Anti-mouse Siglec-F	BD Biosciences	Cat# 562068	E50-2440 (PE)
Anti-mouse CD3	BioLegend	Cat#100311	145-2C11 (APC)
Anti-mouse NK1.1	BioLegend	Cat# 108708	PK136 (PE)
Anti-mouse CD11c	BioLegend	Cat# 117317	N418 (PE/Cyanine7)
NP-PE	Biosearch Technologies	Cat# N-5070-1	Existing entry retained

**Immunoblotting and Co-IP antibodies**			
Anti-BCL6	Abcam	Cat# ab272859	1:5000
Anti-AID	Cell Signaling Technology	Cat# 4975S	1:2000
Anti-CHK1	Abcam	Cat# ab40866	1:2000
Anti-β-actin	Abcam	Cat# ab14748	1:10000
Anti-GLUD1	Invitrogen	Cat# MA573137	1:10000
IgG control for Co-IP	Cell Signaling Technology	Cat# 2729	
Anti-Histone H3	Abcam	Cat# ab176842	1:10000
Anti-H3K27me3	Cell Signaling Technology	Cat# 9733	1:2000
Anti-H3K9 me3	Cell Signaling Technology	Cat# 13969	1:2000
Anti-H3K4 me3	Cell Signaling Technology	Cat# 9751	1:2000
Anti-H3K27ac	Cell Signaling Technology	Cat# 8173	1:2000
Anti-H3K9ac	Cell Signaling Technology	Cat# 9649	1:2000
Anti-Histone H4	Cell Signaling Technology	Cat# 14149	1:2000
Anti-H4K12ac	Cell Signaling Technology	Cat# 13534	1:10000
Anti-pan-acetyl-lysine	Cell Signaling Technology	Cat# 9441	
HRP anti-mouse IgG	Cell Signaling Technology	Cat# 7076	1:20000
HRP anti-rabbit IgG	Cell Signaling Technology	Cat# 7074	1:20000
**ChIP reagents and primers**			
IgG isotype control for ChIP	Cell Signaling Technology	Cat# 2729	
Protein A/G magnetic beads	Vazyme	Cat# PB101-01	
BCL6 ChIP primers	Tsingke	-352 F (CTCGCCCTCGTGTATCCTCT)<br>-352 R (TGGTGGACCTAGGCCATTCT)<br>-217 F (CTCATTTGGCACGCTCCCTA)<br>-217 R (CCTACCATGCCATCAGCACT)<br>0 F (CTGGTGTCCGGCCTTTCCTA)<br>0 R (CCGCAGCTCAAATTCCGAGA)	
**CHIP buffer**			
Cell Lysis Buffer I	In house	50 mM HEPES-KOH pH 7.5<br>140 mM NaCl<br>1 mM EDTA<br>10% glycerol<br>0.5% IGEPAL CA-630<br>0.25% Triton X-100	(working concentration)
Nuclear Lysis Buffer	In house	10 mM Tris-HCl pH 8.0<br>200 mM NaCl<br>1 mM EDTA pH 8.0, 0.5 mM EGTA pH 8.0	(working concentration)
Balance Buffer	In house	10 mM Tris-HCl pH 8.0<br>100 mM NaCl<br>1 mM EDTA pH 8.0, 0.5 mM EGTA pH 8.0<br>0.1% sodium deoxycholate<br>0.5% N-lauroylsarcosine	(working concentration)
qPCR primers	Tsingke	BCL6 F (CCGGCACGCTAGTGATGTT)<br>BCL6 R (TGTCTTATGGGCTCTAAACTGCT)<br>AICDA F (GCCACCTTCGCAACAAGTCT)<br>AICDA R (CCGGGCACAGTCATAGCAC)<br>18S F (CATGCAAACCCACGACAGTA)<br>18S R (CCTCACGCAGCTTGTTGTCTA)<br>ACTB F (ACCGTGAAAAGATGACCCAG)<br>ACTB R (GTACGACCAGAGGCATACAG)	
VH186.2 / VH1-72 SHM primers	Tsingke	Outer F1 (CATGGGATGG AGCTGTATCATGC)<br>Inner F2 (GGTGACAATGACATCCACTTTGC)<br>Outer R1 (CTCACAAGAGTCCGATAGACCCTG)<br>Inner R2 (GAC TGTGAGAGTGGTGCCTTG)	
**Metabolomics and flux**			

UHPLC-MS/MS platform	Metabo-Profile Biotechnology (Shanghai)	ACQUITY-I UPLC / Xevo TQS	
Agilent Seahorse XF Analyzer	Agilent	Model XF96	
Mito Stress Test Kit	Agilent	Cat# 103015-100	
**SPR**			
SPR system	Cytiva	Biacore 8K	
Protein A sensor chip	Cytiva	Cat# 29127556	
HBS-EP+ buffer	Cytiva	Cat# BR-1006-69	
Glycine regeneration solution	in-house	10 mM glycine, pH 1.5	
**Software**			
FlowJo	BD Biosciences / TreeStar	Version 10.8	
GraphPad Prism	GraphPad Software	Version 8.0	
Biacore Insight Evaluation Software	Cytiva	Version 1.0	
