# Supplementary material for: Enhanced SIRT3 activity potentiates germinal center epigenetic modulation through α-ketoglutarate
Source: J Biol Chem. 2026 Sep 12;302(10):113552. doi: 10.1016/j.jbc.2026.113552 (PMC13676980; doi:10.1016/j.jbc.2026.113552)
Supplement: Supplementary file 2 [file mmc2.pdf]

Supplementary Figure 1

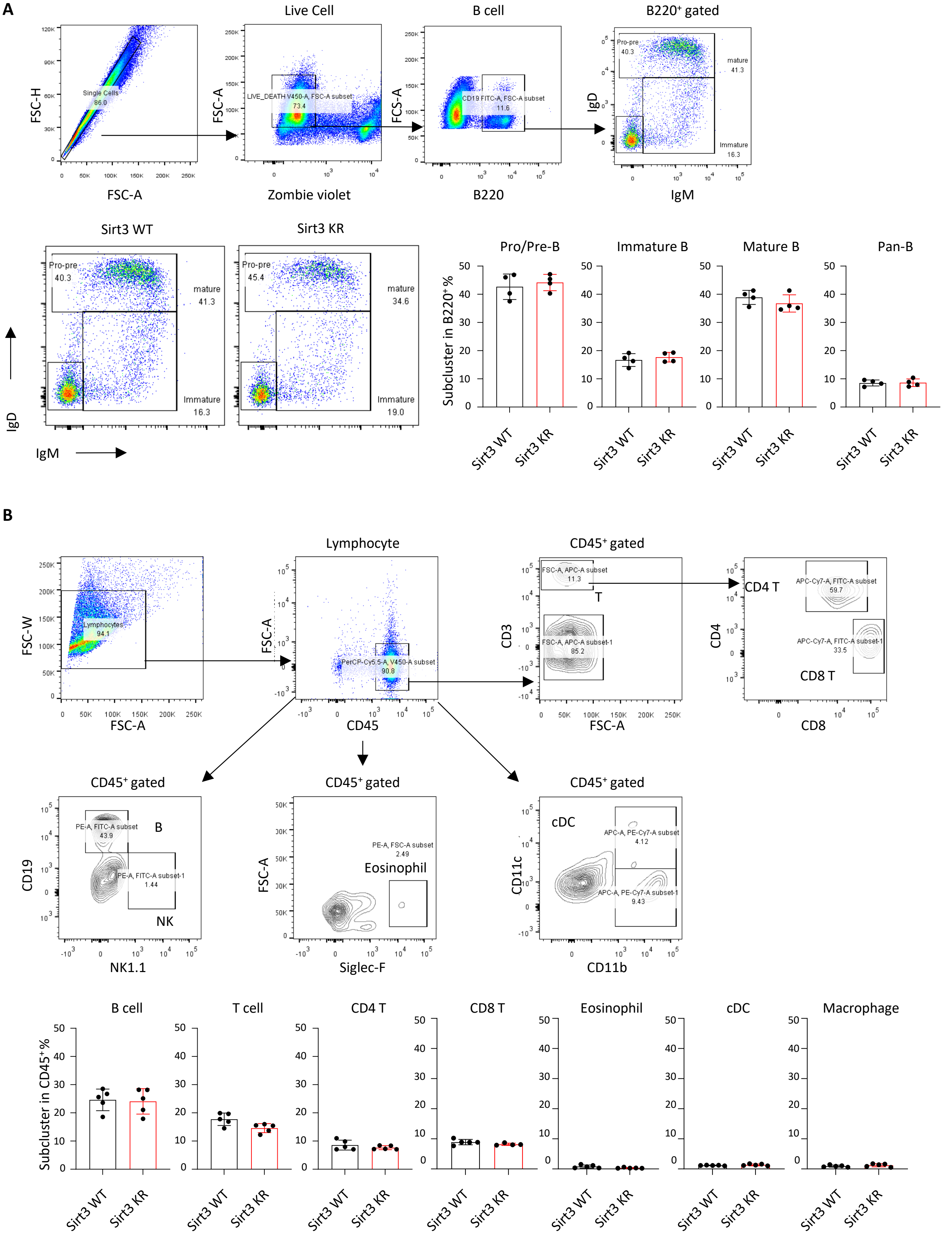

Figure S1. SIRT3 activation does not grossly disrupt basal B cell development or splenic cellularity. (A) Bone marrow B cell developmental stages in SIRT3 wild-type and SIRT3 K223R mice. (B) Major splenic immune cell populations. The gating strategy and Sirt3 WT plot are derived from the same representative experiment. Data are mean ± SD. $^{*}p < 0.05$; $^{**}p < 0.01$; $^{***}p < 0.001$. Two-tailed unpaired Student's t tests were used. n = 4 mice in A; n = 5 mice in B. Data are representative of two independent experiments.
