# Supplementary material for: Enhanced SIRT3 activity potentiates germinal center epigenetic modulation through α-ketoglutarate
Source: J Biol Chem. 2026 Sep 12;302(10):113552. doi: 10.1016/j.jbc.2026.113552 (PMC13676980; doi:10.1016/j.jbc.2026.113552)
Supplement: Supplementary file 3 [file mmc3.pdf]

Supplementary Figure 2

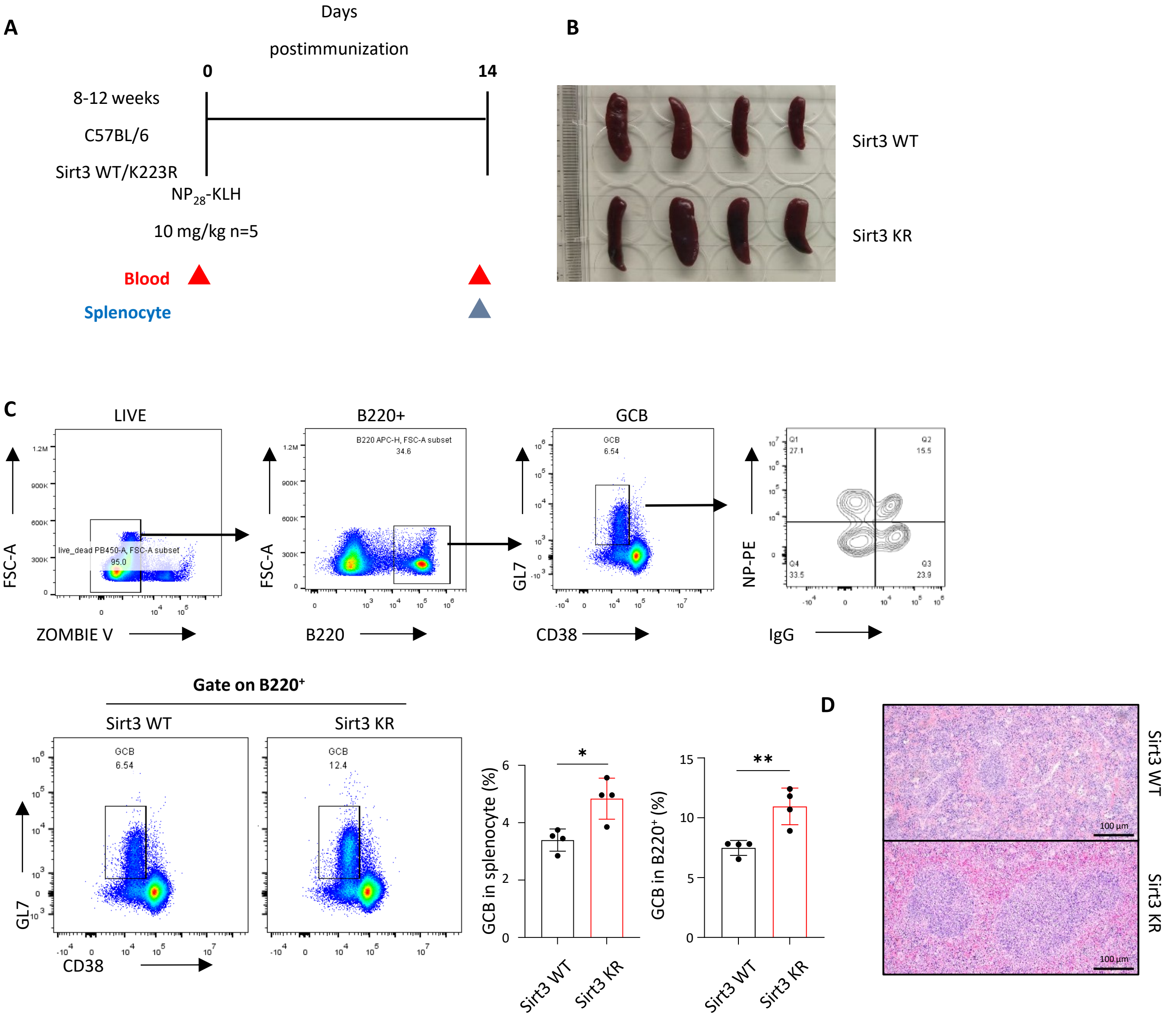


**Figure S2. Antigen-induced germinal center expansion in SIRT3 K223R mice.**
(A) NP28-KLH immunization scheme. (B) Representative spleen morphology. (C) Germinal center B cell quantification. The gating strategy and Sirt3 WT plot are derived from the same representative experiment. (D) Representative H&E-stained spleen sections after immunization. Scale bar, 100 µm. Data are mean ± SD. *$p < 0.05$; **$p < 0.01$; ***$p < 0.001$. Two-tailed unpaired Student's t tests were used. n = 4 mice in C.
