# Supplementary material for: Enhanced SIRT3 activity potentiates germinal center epigenetic modulation through α-ketoglutarate
Source: J Biol Chem. 2026 Sep 12;302(10):113552. doi: 10.1016/j.jbc.2026.113552 (PMC13676980; doi:10.1016/j.jbc.2026.113552)
Supplement: Supplementary file 4 [file mmc4.pdf]

Supplementary Figure 3

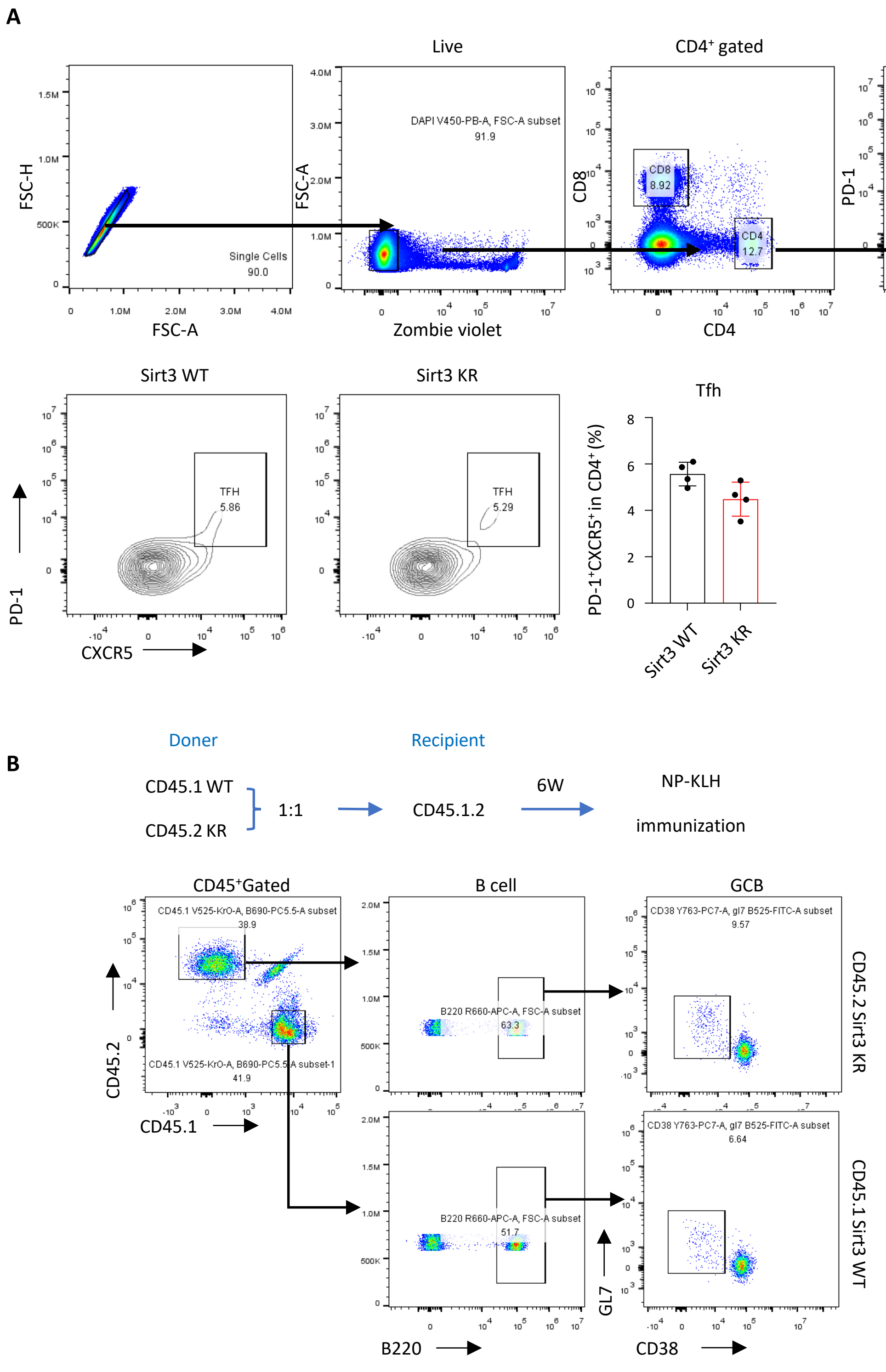

Figure S3. B cell-intrinsic contribution of SIRT3 activation.

(A) T follicular helper cell frequency after immunization. (B) Gating and donor contribution in competitive bone marrow chimeras. Data are mean ± SD. *p < 0.05; **p < 0.01; ***p < 0.001. Two-tailed unpaired Student's t tests were used. n = 4 mice in A; n = 8 mice in B. Data are representative of two independent experiments in A.
