# Supplementary material for: Enhanced SIRT3 activity potentiates germinal center epigenetic modulation through α-ketoglutarate
Source: J Biol Chem. 2026 Sep 12;302(10):113552. doi: 10.1016/j.jbc.2026.113552 (PMC13676980; doi:10.1016/j.jbc.2026.113552)
Supplement: Supplementary file 5 [file mmc5.pdf]

Supplementary Figure 4

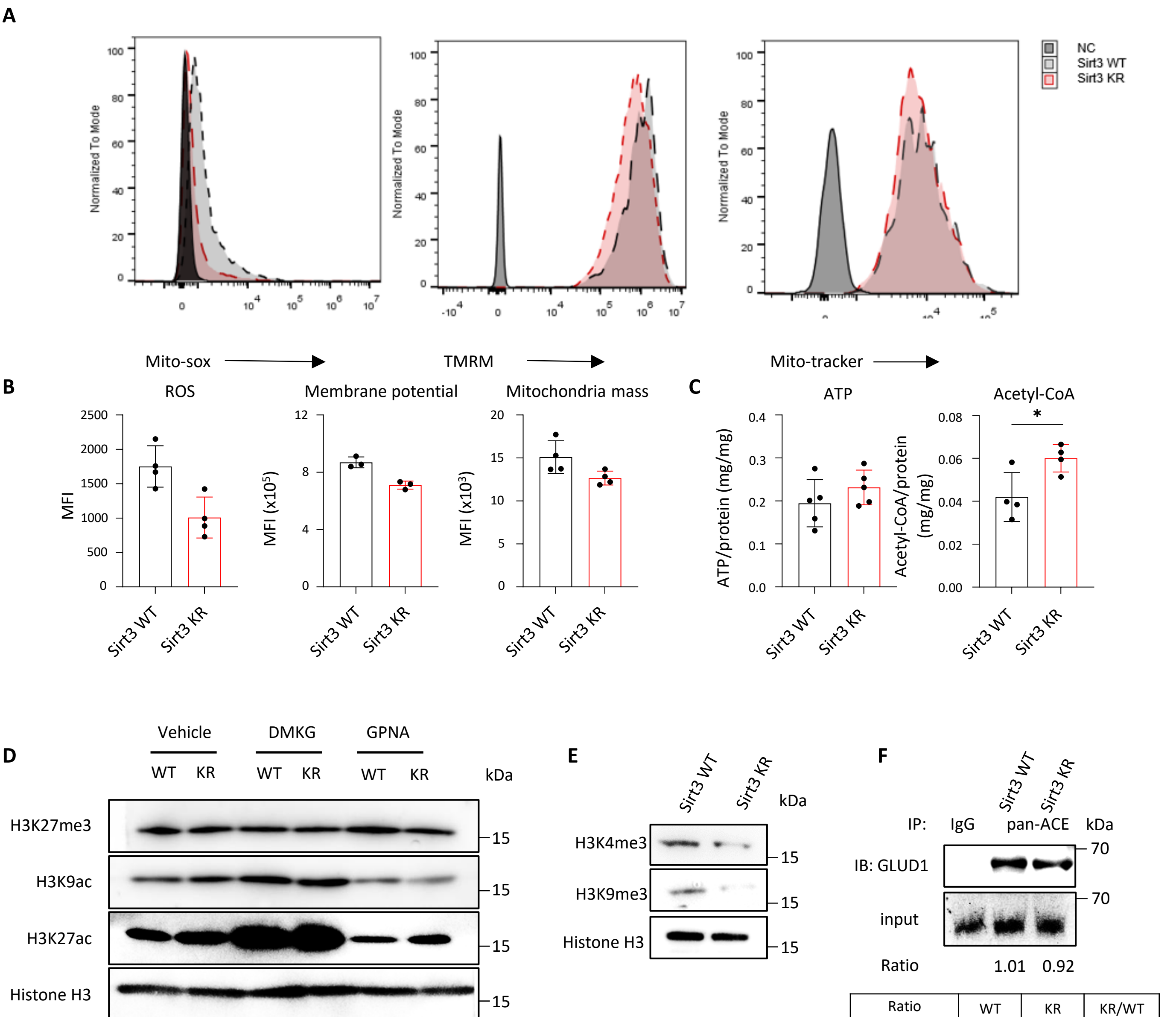

Ratio	WT	KR	KR/WT
REEPAT1	1.01	0.92	0.91
REEPAT2	2.19	0.53	0.24
REEPAT3	0.65	0.41	0.63

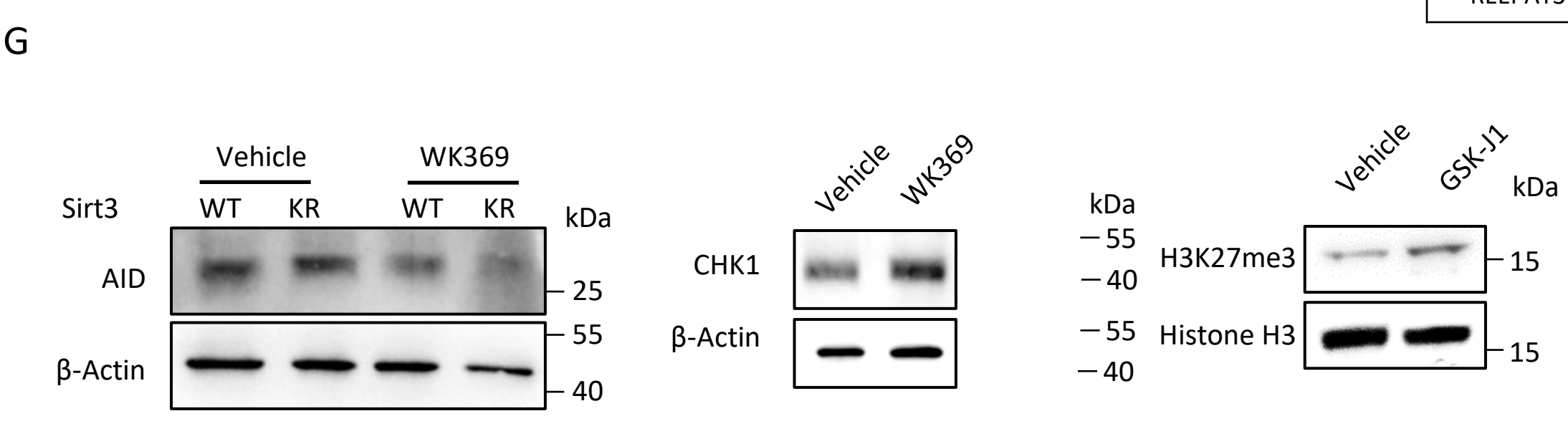

Figure S4. Mitochondrial features of SIRT3 K223R B cells.

(A, B) Mitochondrial reactive oxygen species, membrane potential, and mass. (C) ATP and acetyl-CoA measurements. (D) Histone modification analysis after GPNA or α-ketoglutarate treatment. (E) Histone modification analysis in activated Sirt3 WT and KR B cells. (F) Representative GLUD1 acetylation analysis in activated Sirt3 WT and KR B cells. (G)Protein expression and histone modification analysis after WK-369 or GSK-J1 treatment. Data are mean ± SD. $^{*}p < 0.05$; $^{**}p < 0.01$; $^{***}p < 0.001$. Two-tailed unpaired Student's t tests were used. n = 4 mice in A-C; Data are representative of two independent experiments.
