# Supplementary material for: Enhanced SIRT3 activity potentiates germinal center epigenetic modulation through α-ketoglutarate
Source: J Biol Chem. 2026 Sep 12;302(10):113552. doi: 10.1016/j.jbc.2026.113552 (PMC13676980; doi:10.1016/j.jbc.2026.113552)
Supplement: Supplementary file 6 [file mmc6.pdf]

Supplementary Figure 5

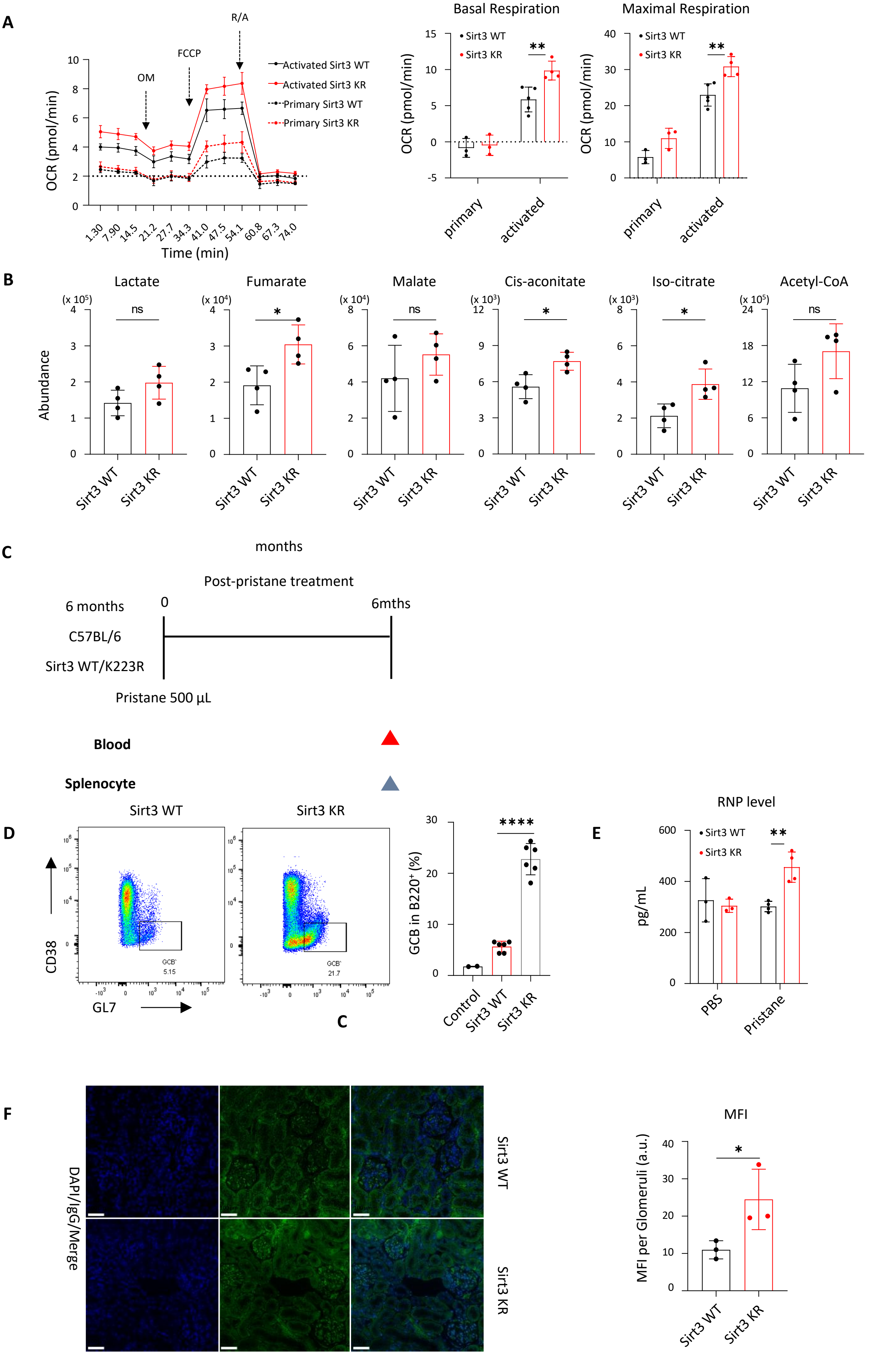

Figure S5. Metabolic and autoimmune consequences of sustained SIRT3 activation.

(A) Seahorse oxygen-consumption analysis. (B) LC-MS/MS measurement of lactate, tricarboxylic acid-cycle intermediates, and acetyl-CoA. (C) Pristane treatment scheme. (D) Germinal center B-cell frequencies in spleens of pristane-treated mice. (E) Serum anti-nuclear antibody levels. (F) Renal IgG deposition after pristane-induced lupus. Scale bar, 100 μm. Renal IgG mean fluorescence intensity was quantified across multiple randomly selected glomeruli from three mice per genotype and analyzed using a mixed-effects model with glomeruli nested within mice. Data are mean ± SD. *$p < 0.05$; **$p < 0.01$; ***$p < 0.001$. Two-tailed unpaired Student's t tests were used in B, one-way ANOVA were used in D, two-way ANOVA were used in A, E. n =3-5 mice in A, n = 4 mice in B, n = 5 or 6 mice in D, n = 3 or 4 mice in E, one animal from the KR group was excluded from renal IgG quantification due to technical issues during tissue sectioning/immunostaining.
