# Supplementary material for: Enhanced SIRT3 activity potentiates germinal center epigenetic modulation through α-ketoglutarate
Source: J Biol Chem. 2026 Sep 12;302(10):113552. doi: 10.1016/j.jbc.2026.113552 (PMC13676980; doi:10.1016/j.jbc.2026.113552)
Supplement: Supplementary file 7 [file mmc7.pdf]

Supplementary Figure 6

A

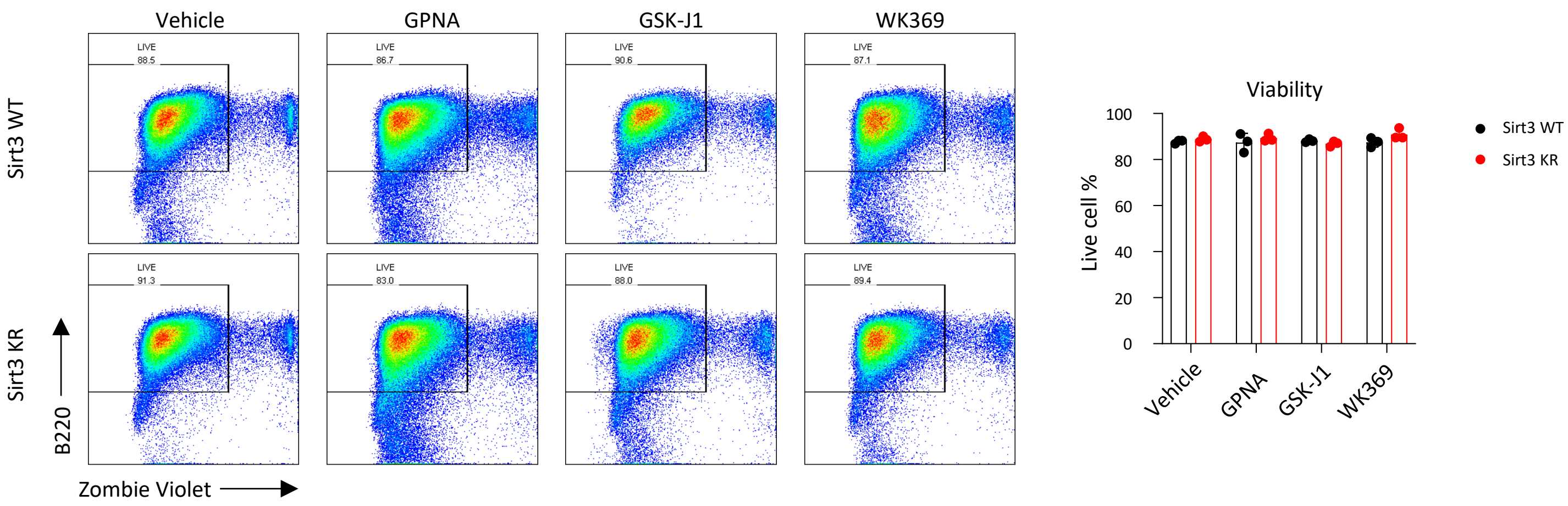

B

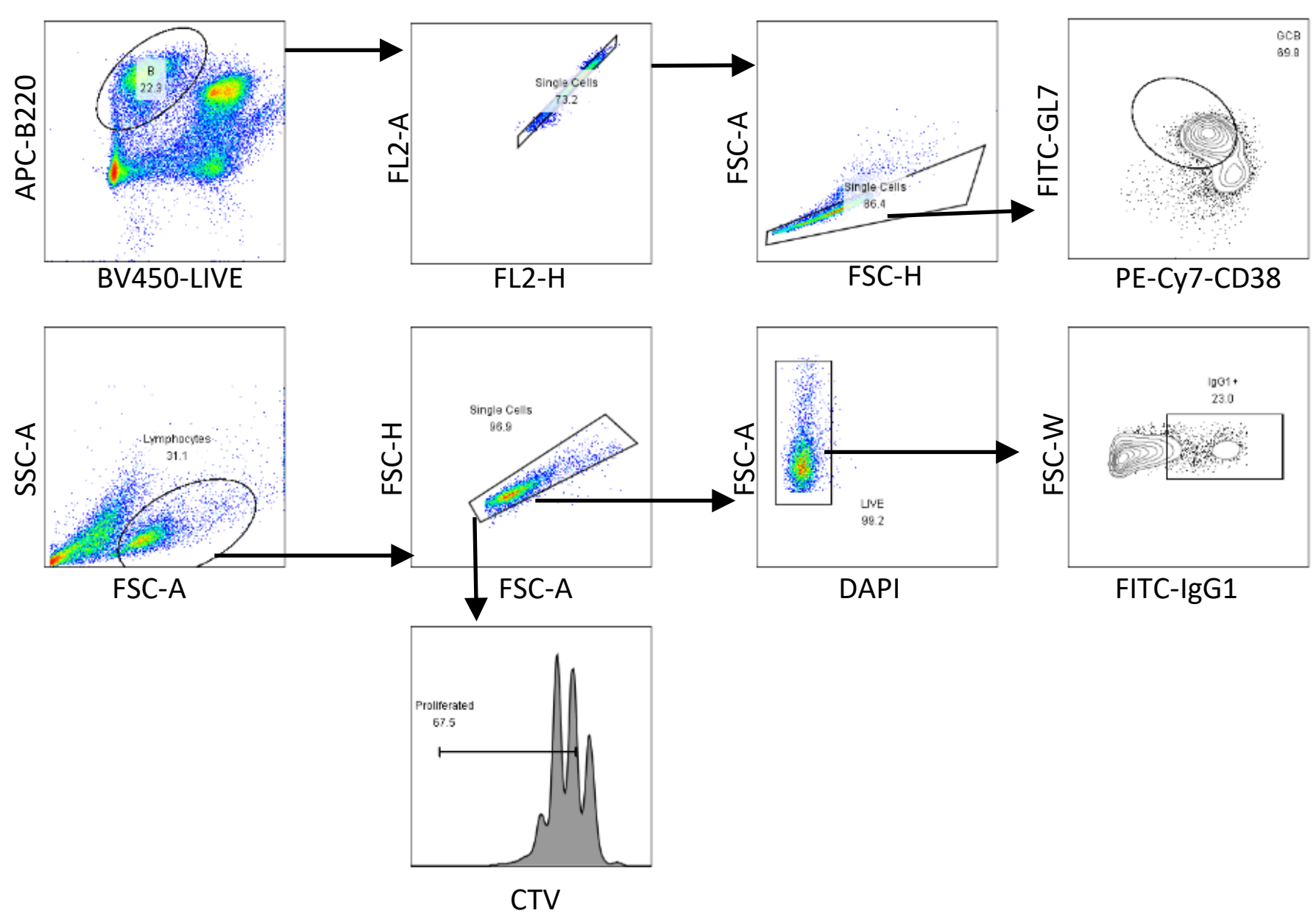

Figure S6. Assessment of cell viability after inhibitor treatment.

(A) B cells were treated with GPNA (10 µM), GSK-J1 (10 µM), or WK-369 (10 µM) under the same conditions as in Figures 4F and 4G, and viability was determined by Zombie Violet exclusion using flow cytometry. (B) Gating strategy for invitro activated systems. Data are mean ± SD from three independent experiments. By two-way ANOVA, None of the treatments significantly altered viability relative to vehicle controls ($P > 0.05$). Data are mean ± SD. *$p < 0.05$; **$p < 0.01$; ***$p < 0.001$.
